# Understanding Precatalyst Activation and Speciation in Manganese-Catalyzed C–H Bond Functionalization Reactions

**DOI:** 10.1021/acs.organomet.3c00004

**Published:** 2023-04-03

**Authors:** Jonathan B. Eastwood, L. Anders Hammarback, Thomas J. Burden, Ian P. Clark, Michael Towrie, Alan Robinson, Ian J. S. Fairlamb, Jason M. Lynam

**Affiliations:** † Department of Chemistry, University of York, Heslington, York YO10 5DD, United Kingdom; ‡ Central Laser Facility, Research Complex at Harwell, STFC Rutherford Appleton Laboratory, Harwell Campus, Didcot, Oxfordshire OX11 0QX, United Kingdom; § Syngenta Crop Protection AG Schaffhauserstrasse, 4332 Stein, Switzerland

## Abstract

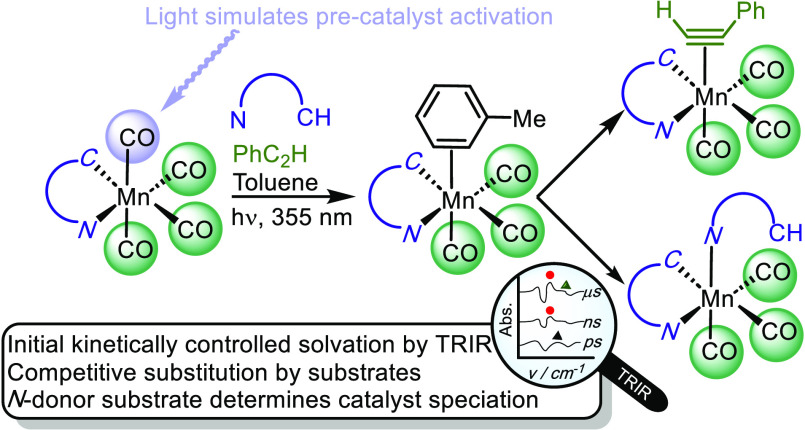

An investigation into species formed following precatalyst activation in Mn-catalyzed C–H bond functionalization reactions is reported. Time-resolved infrared spectroscopy demonstrates that light-induced CO dissociation from precatalysts [Mn(C^N)(CO)_4_] (C^N = cyclometalated 2-phenylpyridine (**1a**), cyclometalated 1,1-bis(4-methoxyphenyl)methanimine (**1b**)) in a toluene solution of 2-phenylpyridine (**2a**) or 1,1-bis(4-methoxyphenyl)methanimine (**2b**) results in the initial formation of solvent complexes *fac*-[Mn(C^N)(CO)_3_(toluene)]. Subsequent solvent substitution on a nanosecond time scale then yields *fac*-[Mn(C^N)(CO)_3_(κ^1^-(*N*)-**2a**)] and *fac*-[Mn(C^N)(CO)_3_(κ^1^-(*N*)-**2b**)], respectively. When the experiments are performed in the presence of phenylacetylene, the initial formation of *fac*-[Mn(C^N)(CO)_3_(toluene)] is followed by a competitive substitution reaction to give *fac*-[Mn(C^N)(CO)_3_(**2**)] and *fac*-[Mn(C^N)(CO)_3_(η^2^-PhC_2_H)]. The fate of the reaction mixture depends on the nature of the nitrogen-containing substrate used. In the case of 2-phenylpyridine, migratory insertion of the alkyne into the Mn–C bond occurs, and *fac*-[Mn(C^N)(CO)_3_(κ^1^-(*N*)-**2a**)] remains unchanged. In contrast, when **2b** is used, substitution of the η^2^-bound phenylacetylene by **2b** occurs on a microsecond time scale, and *fac*-[Mn(C^N)(CO)_3_(κ^1^-(*N*)-**2b**)] is the sole product from the reaction. Calculations with density functional theory indicate that this difference in behavior may be correlated with the different affinities of **2a** and **2b** for the manganese. This study therefore demonstrates that speciation immediately following precatalyst activation is a kinetically controlled event. The most dominant species in the reaction mixture (the solvent) initially binds to the metal. The subsequent substitution of the metal-bound solvent is also kinetically controlled (on a ns time scale) prior to the thermodynamic distribution of products being obtained.

## Introduction

One of the most important steps in transition-metal-catalyzed reactions is the activation of the precatalyst. In most reactions catalyzed by organometallic complexes, the bench-stable reagent added to a reaction is a precatalyst that requires activation before it can participate in the bond activation and formation events that constitute the catalytic reaction coordinate. This activation process may arise from a number of different pathways. For example, a saturated precatalyst may undergo the loss of a coordinated ligand to permit substrate binding, or alternatively, a change in the coordination mode of an already coordinated ligand may promote the same phenomenon. Other examples of precatalyst activation include a change in oxidation state (*e.g.*, Pd(II) → Pd(0)) or the use of a hemilabile ligand to reveal a vacant coordination site.

In all cases, the resulting activated metal complex is more reactive than the precatalyst and thus may initially interact with many of the different reaction components rather than the desired substrate alone. Gaining insight into the immediate fate of an activated precatalyst and its interaction with the different components of the reaction mixture is challenging, primarily due to its anticipated high reactivity and commensurately short lifetime.

This problem is exacerbated when studying the chemistry of 3d transition metal catalysts. Due to the smaller radial extent of the 3d orbitals, metal–ligand bonds are generally weaker compared to their 4d and 5d cogeners.^1^ Furthermore, metal complexes based on 3d metals are more prone to one-electron, rather than two-electron, transfer reactions,^2^ and different preferential coordination numbers entail that different mechanistic pathways with higher rates of substitution may occur.^3^ Although care must be taken with such generalities (for example, the rate constant of substitution of M(CO)_5_(THF) by donor ligands increases in the order Mo > Cr > W with a shift in mechanism from dissociative to associative as the periodic table is descended),^4^ it is clear that the mechanistic processes underpinning catalysis by 3d metals have the potential for increased complexity.^5^

In a series of recent studies, we have demonstrated how time-resolved infrared (TRIR) spectroscopy may be used to directly observe the key steps underpinning Mn-catalyzed C–H bond functionalization reactions.^6−12^ Central to the success of this approach has been the light-induced loss of a carbonyl ligand from a precatalyst, [Mn(C^N)(CO)_4_] (C^N = cyclomanganated ligand). The loss of CO correlates with the activation pathway observed under thermal conditions to give the catalytically active tricarbonyl complexes, *fac*-[Mn(C^N)(CO)_3_(L)].^13−15^

**Figure 1:**
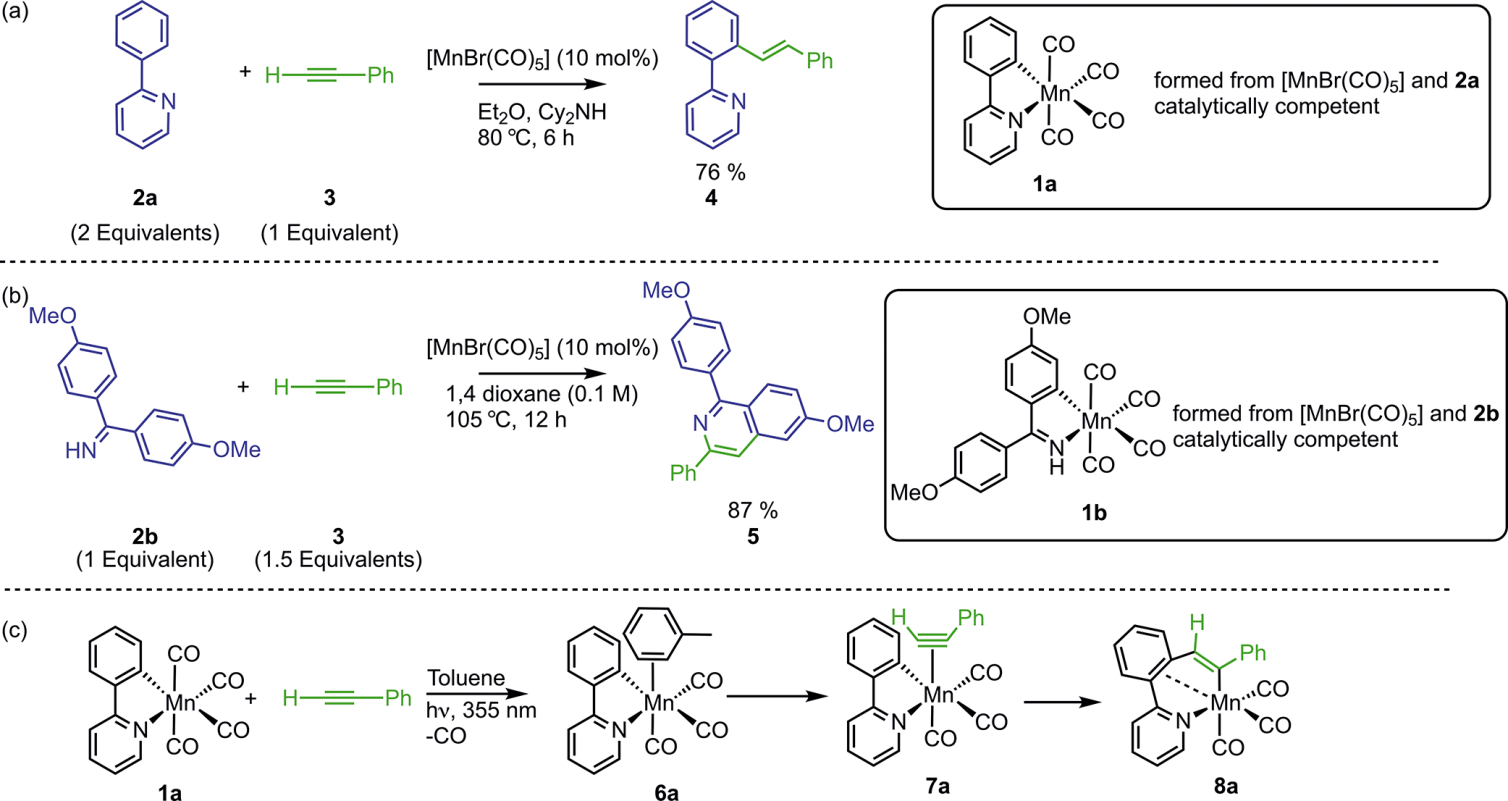
(a) Manganese-catalyzed alkenylation of 2-phenylpyridine. (b) Manganese-catalyzed [4 + 2] annulation reaction. (c) Previously reported observation of light-induced CO loss, solvation, alkyne binding, and insertion into the Mn–C bond.

In the TRIR experiments, the photochemical pump simulates the activation process occurring thermally, and the subsequent IR probe pulse allows the fate of the manganese complex to be monitored through the vibrational modes of the remaining carbonyl ligands. The experiments have harnessed the ability of the time-resolved multiple-probe spectroscopy (TR^M^PS) method^16,17^ to acquire spectra with pump–probe delays ranging from picoseconds to milliseconds. This has allowed for the observation of (i) solvent-coordinated complexes following CO loss;^7^ (ii) coordination and subsequent insertion of alkynes, alkenes, and isocyanates into the Mn–C bond^6,11,12,18^ (Figure 1a); (iii) proton-shuttling events with the coordination sphere of the metal, including the microscopic reverse of the concerted metalation–deprotonation (CMD) mechanism;^10^ (iv) competition between water and N_2_ ligands for the manganese;^8^ and (v) the intermediates involved in the borylation of aryl and heteroaryl diazonium salts.^9^

It was anticipated that this approach could be used to gain an understanding of the speciation that occurs following the activation of the [Mn(C^N)(CO)_4_] precatalyst in catalytic reaction mixtures. The band positions of the photoproducts arising from CO loss from [Mn(C^N)(CO)_3_(L)] are highly sensitive to the nature of the newly coordinated ligand “L” and the spectroscopic resolution of the LIFEtime spectrometer at the ULTRA facility used for the TR^M^PS experiments (*ca.* 2 cm^–1^), meaning that complex spectra containing multiple species may be deconvoluted. A strategy was envisaged in which the interactions of light-activated [Mn(C^N)(CO)_4_] with the different components of a catalytic reaction could be investigated separately and the nature and dynamics of the resulting photoproducts investigated in isolation. Experiments would then be performed on catalytic reaction mixtures, and insight into the speciation of the activated complex would be obtained by comparison to the reference spectrum of each component. These data would therefore enable the immediate fate of the manganese complex upon activation to be determined. Such insight is especially important considering the recent report by Larrosa and co-workers which demonstrated that *fac*-[MnBr(CO)_3_(NCMe)_2_] is a viable catalyst for room-temperature Mn-catalyzed C–H bond functionalization.^19^ This demonstrates the importance of complexes containing a “Mn(CO)_3_” moiety in these reactions: our light-induced strategy allows for direct access to this structural unit.

The successful demonstration of this strategy is now reported. These experiments demonstrate that in all cases initial coordination of the solvent occurs, followed by substitution in an essentially statistical manner by the other components of the reaction. The subsequent fate of the complexes then depends on the nature of the *N*-donor ligand employed.

## Results and Discussion

Two archetypical Mn-catalyzed reactions were selected for study. In the first instance, the reaction between 2-phenylpyridine (**2a**) and phenylacetylene (**3**) to give alkenylated product **4** was explored. As shown by Wang,^14^ the cyclomanganated 2-phenylpyridine complex **1a** was a viable precatalyst for this reaction (Figure 1a). Second, the reaction between 1,1-bis(4-methoxyphenyl)methanimine (**2b**) and **3** to afford isoquinoline **5** was investigated (Figure 1b).^20^ In this case the catalytically competent cyclomanganated imine complex **1b** was used as a precursor.

Our procedure to explore the chemistry of these systems is based on a TRIR spectroscopy experiment. Here solutions of the complexes are continuously flowed through an IR cell that is held in the path of overlapped pump and probe beams. The solution is continuously replenished, as the photochemistry described in this work is irreversible and hence fresh sample is continually required. The pump pulse (λ = 355 nm) induces loss of CO from **1a** and **1b**.

The subsequent changes to speciation are then followed by a probe pulse that interrogates the IR spectrum of the sample between *ca*. 1850 and 2100 cm^–1^. The probe pulse arrives at a defined time following activation by the pump, and this interval between pulses is referred to as the pump–probe delay, *t*. The synchronization provided by TR^M^PS means that this delay is repeated every 10 μs, and therefore, for every pump pulse spectra are recorded at *t*, *t* + 10 μs, *t* + 20 μs, *t* + 30 μs, *etc.* for 990 μs. The resulting data are presented as difference spectra with negative peaks corresponding to material consumed upon photolysis (in all cases, these correspond to the ground-state IR spectrum of the appropriate complex **1**) and positive peaks representing the newly formed photoproducts.

It is important to highlight that irradiation of complex **1a** results in competitive formation of ^3^[**1a**] and solvent complexes *fac*-[Mn(ppy)(CO)_3_(S)]. The electronically excited state ^3^[**1a**] has a lifetime of *ca*. 5 ps. At short pump–probe delays, vibrationally excited *fac*-[Mn(ppy)(CO)_3_(S)] is observed, which has a lifetime of <50 ps.^7^ This work is focused on the chemistry of the photoproducts present at pump–probe delays with *t* > 1 ns, which are therefore in their ground electronic and vibrational states.

**Figure 2:**
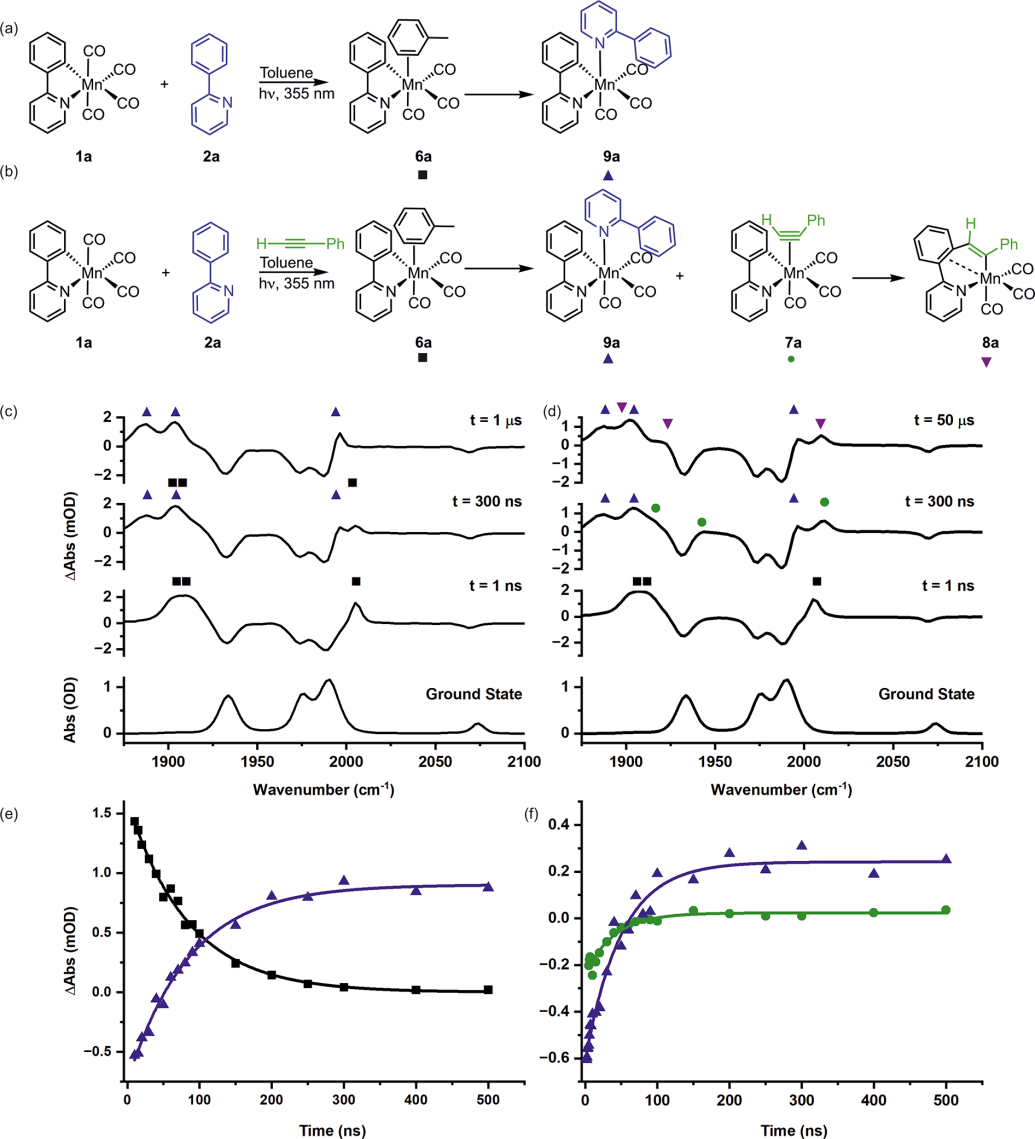
(a) Reaction scheme showing the products formed after photolysis of **1a** in a toluene solution of **2a**. (b) Reaction scheme showing the products formed after photolysis of **1a** in a toluene solution of **2a** and **3**. (c) Ground-state IR spectrum of **1a** in toluene solution (bottom) and TRIR spectra of **1a** in a toluene solution of **2a** at pump–probe delays of 1 ns, 300 ns, and 1 μs. (d) Ground-state IR spectrum of **1a** in toluene solution (bottom) and TRIR spectra of **1a** in a toluene solution of 2-phenylpyridine and phenylacetylene at pump–probe delays of 1 ns, 300 ns, and 50 μs. (e) Kinetic plot showing the changes in intensity of **6a** (black squares) and **9a** (blue triangles) for **1a** in a toluene solution of **2a**. The lines show fits to an exponential decay function for **6a** (*k* = (1.19 ± 0.14) × 10^7^ s^–1^, *R*^2^ = 0.99) and a growth function for **9a** (*k* = (1.17 ± 0.17) × 10^7^ s^–1^, *R*^2^ = 0.99). (f) Kinetic plot showing the competitive formation of **7a** (green circles) and **9a** (blue triangles) for **1a** in a toluene solution of **2a** and **3**. The lines show fits to exponential growth functions (for **7a**, *k* = (2.51 ± 0.77) × 10^7^ s^–1^, *R*^2^ = 0.95; for **9a**, *k* = (2.01 ± 0.39) × 10^7^ s^–1^, *R*^2^ = 0.98). The concentrations used in these experiments were [**1a**] = 1.98 mmol dm^–3^, [**2a**] = 232 mmol dm^–3^ in (c) and (e) or 234 mmol dm^–3^ in (d) and (f), and [**3**] = 231 mmol dm^–3^.

In previous studies, the interaction of the light-activated complexes **1a** and **1b** with toluene solutions of PhC_2_H, **3**, was reported.^6,12^ In these cases, the initial formation of toluene complexes (*e.g.*, **6a**; Figure 1c) was followed by substitution by PhC_2_H to give alkyne complexes (*e.g*., **7a**) and finally C–C bond formation to give the seven-membered metallacycles (*e.g*., **8a)**, which are a key intermediates in Mn-catalyzed reactions.^15^ To understand the speciation of the precatalyst following activation via CO loss, the interaction between light-activated **1a** and 2-phenylpyridine in toluene solution was explored using TR^M^PS. The resulting spectra (Figure 2c) demonstrated that at short pump–probe delays (<1 ns) a single species was formed with positive bands at 2005 and 1909 (br) cm^–1^. These were identical to the previously reported toluene complex *fac*-[Mn(C^N)(CO)_3_(toluene)].^7,12^ The coordination geometry at the metal was confirmed by the appearance of the sharp high-energy band and broader feature at lower energy, indicating the pseudo-*C*_3*v*_ symmetry of the complex.^21^

Over the course of *ca.* 500 ns (Figure 2e), the bands for **6a** were replaced by three new features at 1996, 1904, and 1887 cm^–1^, assigned to complex **9a**, a process that obeyed pseudo-first-order kinetics.^22^ On the basis of the shift in CO bands to lower energy, **9a** was assigned as the *N*-bound 2-phenylpyridine complex *fac*-[Mn(C^N)(CO)_3_(κ^1^-(*N*)-**2a**)] (Figure 2a).

The experiment was repeated but with phenylacetylene added to the reaction in equimolar amounts to **2a**. As before, the toluene complex **6a** was the initially formed species, which was then replaced over the course of 100 ns by bands corresponding to both **9a** and alkyne complex **7a** (Figure 2d). The observed rate constants for the formation of **9a**, (2.01 ± 0.39) × 10^7^ s^–1^, and **7a**, (2.51 ± 0.77) × 10^7^ s^–1^, were similar, as should be the case for competitive (pseudo-)first-order reactions.

At longer pump–probe delays, the bands for alkyne complex **7a** were observed to decrease in intensity and to be replaced by those for metallacycle **8a**, formed by migratory insertion into the Mn–C bond. The rate constant, (2.01 ± 0.28) × 10^5^ s^–1^, is similar to those reported previously (Figure 2f).^6,12^ The reaction was performed again, but the ratio of the reagents mirrored those used in a catalytic reaction (**1a** = 0.1 equiv, **2a** = 2 equiv, **3** = 1 equiv). The resulting spectra (see the Supporting Information) demonstrated that the same course of events occurred, with the formation of **8a** (through alkyne complex **7a**) and **9a**. However, in this case, a greater degree of **9a** was formed, as might be expected because **2a** was present in a greater concentration than in the previous experiment. Consequently, **7a** and **8a** were formed in smaller amounts, which entailed that the resulting kinetic information was of lower quality, but the C–C bond formation step was still observed.

Having identified the compounds formed upon activation of a precatalyst relevant to the Mn-catalyzed alkenylation reaction (Figure 1a), a similar series of experiments were performed to determine the fate of **1b**, the precatalyst for the [4 + 2] annulation reaction (Figure 1b).

**Figure 3:**
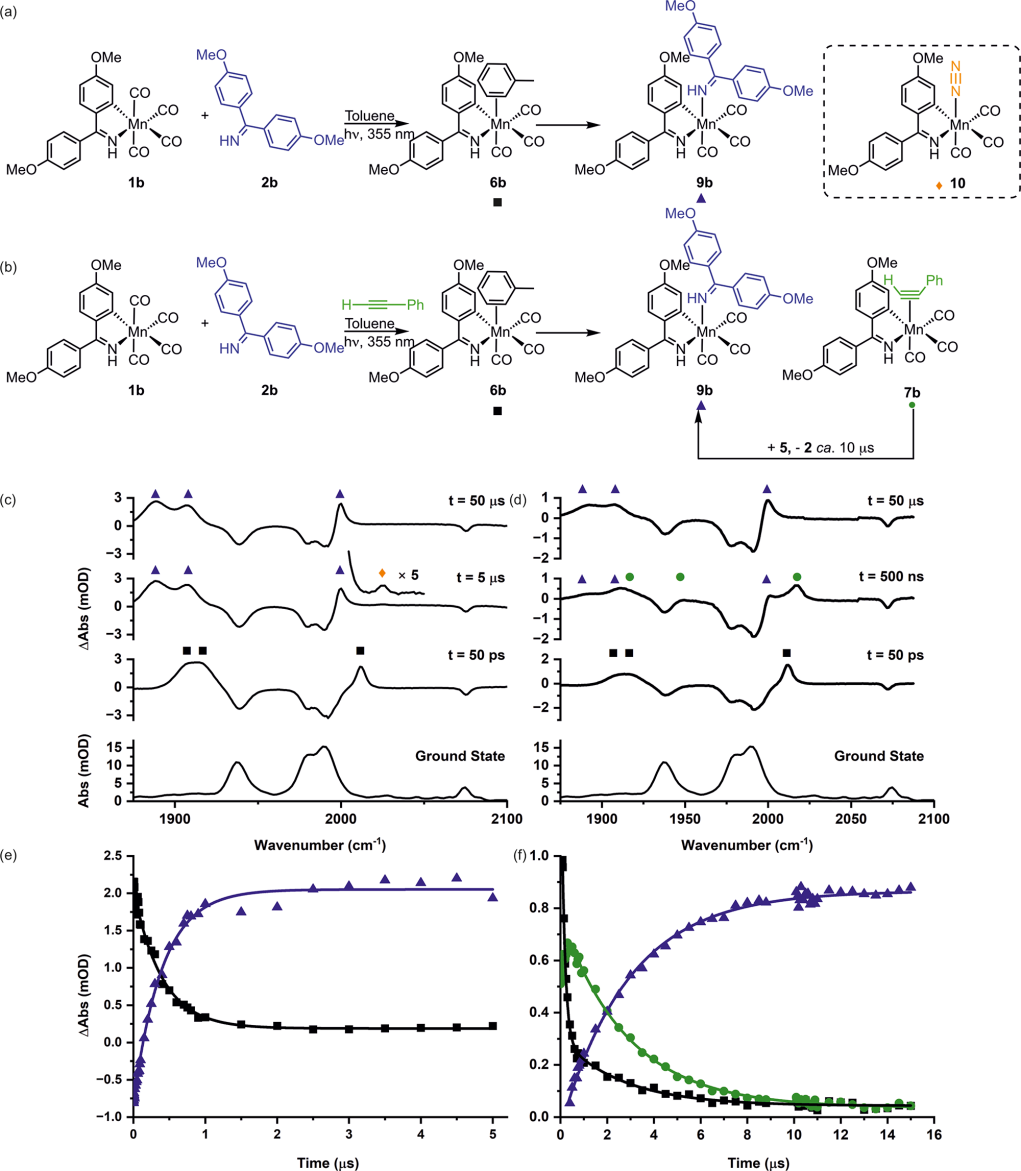
(a) Reaction scheme showing the products formed after photolysis of **1b** in a toluene solution of **2b**. (b) Reaction scheme showing the products formed after photolysis of **1b** in a toluene solution of **2b** and **3**. (c) Ground-state IR spectrum of **1b** in toluene solution (bottom) and TRIR spectra of **1b** in a toluene solution of **2b** at pump–probe delays of 50 ps, 5 μs, and 50 μs. (d) Ground-state IR spectrum of **1b** in toluene solution (bottom) and TRIR spectra of **1b** in a toluene solution of **2b** and **3** at pump–probe delays of 50 ps, 500 ns, and 50 μs. (e) Kinetic plot showing the changes in intensity of **6b** (black squares) and **9b** (blue triangles) for **1b** in a toluene solution of **2b**. The lines show fits to an exponential decay function for **6b** (*k* = (2.53 ± 0.32) × 10^6^ s^–1^, *R*^2^ = 0.99) and a growth function for **9b** (*k* = (2.49 ± 0.18) × 10^6^ s^–1^, *R*^2^ = 0.99). (f) Kinetic plot showing the changes in intensity of **6b** (black squares), **7b** (green circles), and **9a** (blue triangles) for **1a** in a toluene solution of **2b** and **3**. The lines show fits to a biexponential decay function for **6b** (*k*_1_*=* (6.98 ± 0.27) × 10^6^ s^–1^, *k*_2_*=* (3.77 ± 0.74) × 10^5^ s^–1^, *R*^2^ = 0.99), a biexponential growth function for **9b** (*k*_1_*=* (6.11 ± 0.46) × 10^6^ s^–1^, *k*_2_*=* (3.11 ± 0.21) × 10^5^ s^–1^, *R*^2^ = 0.99), and an exponential growth and decay function for **7b** (*k*_1_*=* (9.69 ± 0.29) × 10^6^ s^–1^, *k*_2_*=* (3.47 ± 0.19) × 10^5^ s^–1^, *R*^2^ = 0.99). The concentrations used in these experiments were [**1b**] = 2.00 mmol dm^–3^ and [**2b**] = 22.4 mmol dm^–3^ in (c) and (e) and [**1b**] = 2.03 mmol dm^–3^, [**2b**] = 20.5 mmol dm^–3^, and [**3**] = 30.1 mmol dm^–3^ in (d) and (f).

In the first instance, the interaction between light-activated **1b** and 1,1-bis(4-methoxyphenyl)methanimine (**2b**) was explored. Irradiation of **1b** in a toluene solution of **2b** resulted in the expected photodissociation of a CO ligand and the initial formation of the toluene complex *fac*-[Mn(C^N)(CO)_3_(toluene)] (**6b**) (Figure 3c). Over the course of *ca.* 3 μs, the bands for **6b** were observed to decrease in intensity and to be replaced by three highly red-shifted bands with frequencies of 2000, 1907, and 1888 cm^–1^, which were assigned to **9b**. The band intensities again confirmed the formation of a complex with a pseudo-*C*_3*v*_ coordination geometry. The observed rate constant for the growth of these new features was statistically identical to that for the loss of the band for **6b** (Figure 3), indicating that the new species formed from **6b**. The structure of **9b** was assigned as *fac*-[Mn(C^N)(CO)_3_(κ^1^-(*N*)-**2b**)] on the basis that the substantial red shift in the energies of the three carbonyl bands in **9b** indicated that a strongly donating ligand had been incorporated into the coordination sphere of the metal and that the new transient bands did not correspond to those of the previously identified water complex *fac*-[Mn(C^N)(CO)_3_(OH_2_)].^8^

Simultaneously with the formation of **9b** a small band was observed at 2025 cm^–1^, consistent with the formation of *fac*-[Mn(C^N)(CO)_3_(N_2_)] (**10**),^8^ which then depleted at longer pump–probe delays. This indicates that although the binding of N_2_ to the manganese was kinetically competitive with **2b**, the imine was the thermodynamically preferred ligand.

To understand the speciation when precatalyst **1b** is activated, phenylacetylene was then added to the sample to mirror the 1:1.5 ratio of **2b** and **3** used in the catalytic reactions. Once again, toluene complex **6b** was the initially formed photoproduct, but over the course of *ca*. 500 ns, bands corresponding to both **9b** and the previously observed^12^ alkyne complex *fac*-[Mn(C^N)(CO)_3_(η^2^-HC≡CPh)] (**7b**) appeared.

The reaction mixture changed at longer pump–probe delays. Over the course of 16 μs the bands for alkyne complex **7b** were observed to decrease in intensity, but no evidence for the previously observed product of alkyne migration into the Mn–C bond of the cyclomanganated imine complex, **8b**, was obtained. Instead, the bands for **9b** continued to grow in intensity (*k* = (3.11 ± 0.21) × 10^5^ s^–1^) with essentially the same rate constant as for the loss of **7b** (*k* = (3.47 ± 0.19) × 10^5^ s^–1^). At long pump–probe delays, **9b** was the only photoproduct observed. Therefore, under these conditions, no evidence of the C–C bond formation step through migratory insertion of the alkyne into the Mn–C bond was obtained. Instead, the coordinated alkyne was substituted by free **2b**. Experiments performed with **1a** and **2a** under identical concentrations (see the Supporting Information) demonstrated that the migratory insertion reaction was still observed, demonstrating that the differences observed in the case of **1b** and **2b** were due to the nature of the substrates used rather an artifact of the conditions.

**Figure 4:**
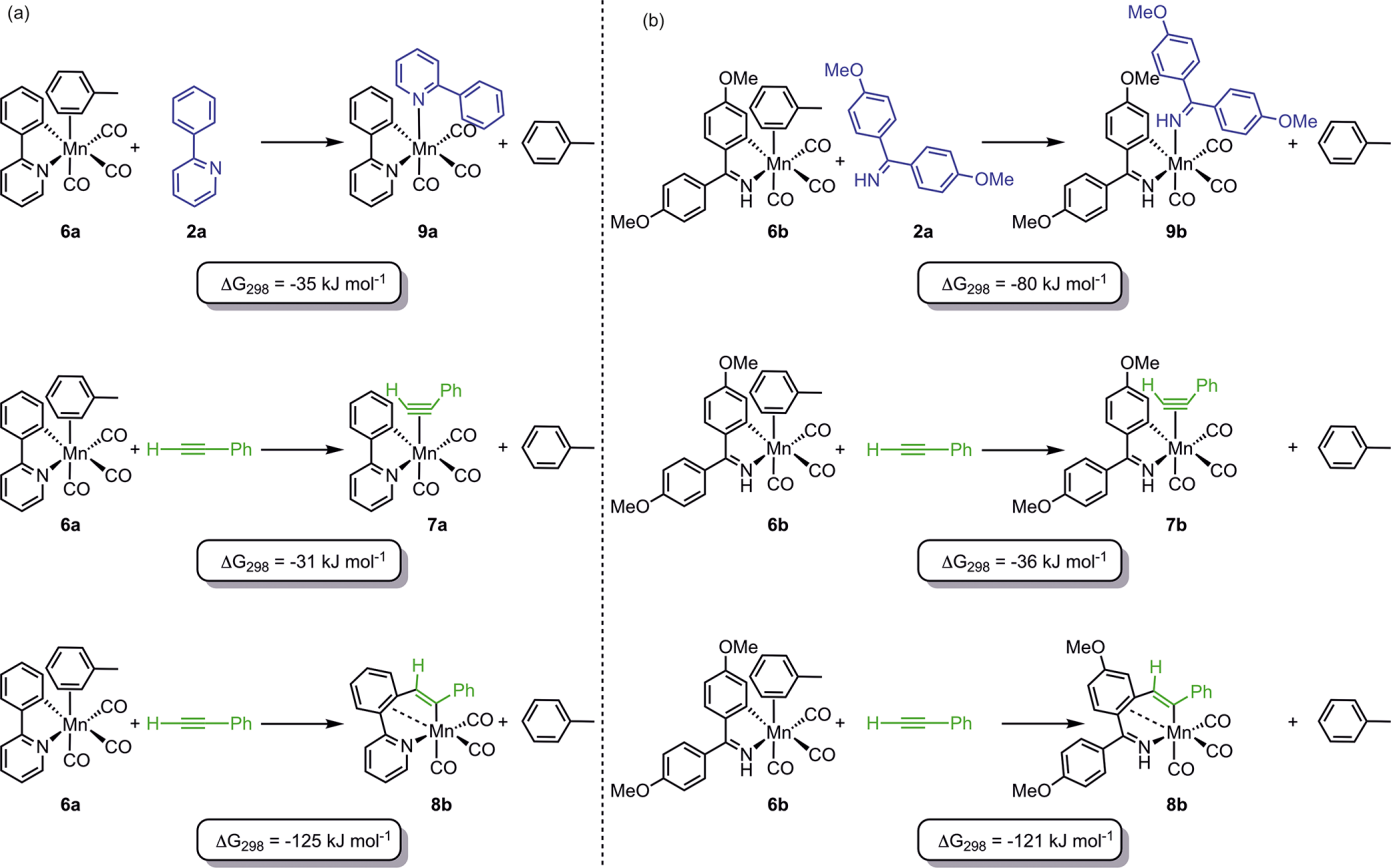
Calculated energy changes upon ligand substitution for (a) 2-phenylpyridine-substituted and (b) 1,1-bis(4-methoxyphenyl)methanimine-substituted complexes. Energies are Gibbs energies at 298 K calculated at the D3-PBE0/def2-TZVPP//BP86/SV(P) level of theory with COSMO solvation in toluene.

A series of calculations using density functional theory (DFT) were performed in order to understand the difference in behavior between the 2-phenylpyridine- and 1,1-bis(4-methoxyphenyl)methanimine-based systems. Details of the methodology used are provided in the Supporting Information. A series of isodesmic reactions were calculated in order to model the change in Gibbs energy for the conversion of **6** to **7**, **8**, and **9** as a function of the nitrogen donor ligand (Figure 4). The resulting data showed a significant change between the two nitrogen donor ligands. In the case of the 2-phenylpyridine substrate, the substitution of the coordinated toluene ligand in **6a** by either **2a** or PhC_2_H was shown to be exergonic by 35 and 31 kJ mol^–1^, respectively. As shown in our previous work, the complex arising from the subsequent alkyne insertion into the metal–carbon bond lies at lower energy than the corresponding alkyne complex.^6,12^

Identical calculations on the 1,1-bis(4-methoxyphenyl)methanimine system provided insight into the difference between the two systems. In this case, the substitution of toluene from **6b** by either **2b** or PhC_2_H was shown to be exergonic by 80 and 36 kJ mol^–1^, respectively. The complex arising from alkyne insertion, **8b**, was still located at −121 kJ mol^–1^ relative to **6b**, although this was not observed when experiments were performed in the presence of **2b**.

It is therefore proposed that the difference in behavior between the two systems reflects the change in relative energy of the alkyne complexes **7** and the *N*-donor complexes **9**. In the case of the imine-based substrate **2b**, the resulting *N*-bound complex **9a** is at a substantially lower energy than the alkyne complex **7b**. Therefore, after the initial kinetically controlled competitive substitution of the toluene in **6b** to give a mixture of **7b** and **9b**, a second ligand substitution occurs in which **2b** replaces the coordinated alkyne to give **9b** as the sole product of the reaction. At the concentrations employed, this is faster than the migratory insertion reaction, as **8b** is the lowest-energy species calculated. Indeed, the observed rate constant for the formation of **9b** from **7b**, (5.1 ± 0.2) × 10^5^ s^–1^, is twice the reported first-order rate constant for the migratory insertion reaction **7b** → **8b**, (2.25 ± 0.16) × 10^5^ s^–1^.^12^

In the case of the 2-phenylpyridine-based system, there is no energetic driving force for **2b** to substitute the phenylacetylene in **9a**: their calculated Gibbs energies are very similar. Hence, no ligand substitution would be expected (as observed experimentally), and migratory insertion occurs.

## Conclusions

The results from this study have demonstrated that the formation of a solvent complex immediately follows the key step in precatalyst activation: ligand dissociation. This is presumably a kinetically controlled event, as the solvent is the dominant component of the reaction mixture. The toluene is a weakly bound ligand, and the second-order rate constant for this substitution is *ca.* 10^7^ mol^–1^ dm^3^ s^–1^. It is therefore proposed that the near-diffusion-controlled substitution of the coordinated solvent is essentially governed by the relative concentrations of the two substrates in the reaction.

In these aspects the photoproducts derived from both **1a** and **1b** behave in an essentially identical manner, but there is a difference in behavior at longer times between the interactions involving 2-phenylpyridine and 1,1-bis(4-methoxyphenyl)methanimine. In the former case, the alkyne complex undergoes the expected migratory insertion reaction to give **8a**. In the latter, the alkyne is substituted by uncoordinated **2b**, and **9b** is the sole product from the reaction. The calculations indicate that this is an artifact of the different affinities of **2a** and **2b** for the manganese, which reflects the thermodynamic preference for the imine-based ligand to act as an effective *N*-donor ligand.

Although at this stage it is not possible to extrapolate these results to demonstrate exactly why the outcome of the Mn-mediated reactions of **2a** and **2b** with alkynes give different outcomes (alkenylation versus annulation in Figure 1a and Figure 1b), the data do demonstrate that the nature of the substrate containing the heteroatom directing group plays a key role in determining catalyst speciation. These results also indicate that the formation of species such as **9** may represent off-cycle catalyst sinks, as loss of the *N*-bound heteroatom-based substrates is required prior to the coordination of the unsaturated substrate and should be considered as part of the mechanistic processes underpinning Mn(I)-catalyzed reactions.

## Supporting Information Available

Cartesian coordinates (XYZ)
Additional TRIR spectra, details of computational chemistry methods, collated rate constants, and calculated energies (PDF)

## Supplementary Material

om3c00004_si_001.xyz
