# Supplementary material for: Understanding Precatalyst Activation and Speciation in Manganese-Catalyzed C–H Bond Functionalization Reactions
Source: Organometallics. 2023 Apr 3;42(14):1766–73. doi: 10.1021/acs.organomet.3c00004 (PMC10369674; doi:10.1021/acs.organomet.3c00004)
Supplement: Supplementary file 2 — om3c00004_si_002.pdf [file om3c00004_si_002.pdf]

Electronic Supporting Information for

Understanding Pre-catalyst Activation and Speciation in Manganese-catalyzed C–H Bond Functionalization Reactions.

Jonathan B. Eastwood,[a] L. Anders Hammarback,[a] Thomas J. Burden,[a] Ian P. Clark,[b] Michael Towrie,[b] Alan Robinson,[c] Ian J. S. Fairlamb*, [a] and Jason M. Lynam*, [a]

[a] Department of Chemistry, University of York, Heslington, York, YO10 5DD, UK ian.fairlamb@york.ac.uk, jason.lynam@york.ac.uk

[b] Central Laser Facility, STFC Rutherford Appleton Laboratory, Harwell Science and Innovation Campus, Didcot, Oxfordshire, OX11 0QX, UK.

[c] Syngenta Crop Protection AG Schaffhauserstrasse, 4332 Stein, Switzerland.

Table of Contents

1 Synthetic Methodology 3
1.1 General Considerations 3
2 TRIR Spectroscopy 3
2.1 Methodology 3
2.2 Compound Numbering 5
2.3 Additional TRIR studies on 1a 6
2.3.1 TRIR study of **1a** in toluene solution with a 2:1 ratio of PhC_2H and 2-ppy. 6
2.3.2 TRIR study of **1a** in toluene solution with a 1:1 ratio of PhC_2H and 2-ppy. 7
2.3.3 TRIR study of **1a** in toluene solution with a 1:1 ratio of PhC_2H and 2-ppy. 8
2.4 Additional TRIR studies on 1b 9
2.4.1 TRIR study of **1b** in toluene solution – 1 9
2.4.2 TRIR study of **1b** in toluene solution – 2 11
2.4.3 Collated Rate Constants 13
3 DFT Calculations 14
3.1 Methodology 14
4 References 16

1 Synthetic Methodology

1.1 General Considerations

Solvents and Reagents

Commercially sourced reagents were purchased from Acros Organics, Alfa Aesar, Fluorochem, or Sigma-Aldrich and used as received unless otherwise stated. Complexes **1a**, **1b** and compound **2b** were prepared as described previously.[1,2]

Infrared Spectroscopy

Infrared spectra were obtained using a Unicam Research Series FTIR (KBr IR) or a Bruker ALPHA-Platinum FTIR Spectrometer with a platinum−diamond ATR sampling module.

2 TRIR Spectroscopy

2.1 Methodology

Time-resolved infra-red spectra were recorded on either the ULTRA A or LIFEtime instrument in the ULTRA facility at the Rutherford Appleton Laboratories using the Time-Resolved Multiple Probe Spectroscopy mode of operation, (hereafter TR^{M}PS).[3]

The experiments on ULTRA A were driven by a 10 kHz repetition rate Ti:Sapphire amplifier (Thales) as a probe source, producing 40 fs pulses at 800 nm. The Ti:Sapphire laser output was used to pump an Optical Parametric Amplifier (TOPAS, Light Conversion Ltd.) followed by a AgGaS Difference Frequency Mixing stage which produced tuneable mid-IR probe beam of ~ 500 cm^{-1} useable bandwidth. The IR probe beam was split to form reference and probe beams, which were passed through spectrographs onto MCT array detectors (IR Associates). The probe beam spot size at sample was ca. 80x80 µm^{2}. High speed data acquisition systems (Quantum Detectors) allowed 10 kHz acquisition and processing of the probe and reference pulses to generate a pump-on pump-off infrared absorption difference signal. The excitation source for the TRIR experiments was the output of the 1 kHz titanium sapphire amplifier (Spectra Physics Spitfire XP, 100 fs pulse length) equipped with another TOPAS OPA, pulse energy at sample attenuated down to 1 µJ and focused down to ca. 150 × 150 µm^{2} spot). Both ULTRA amplifier and Spitfire amplifier were optically synchronised by sharing the same seed from 68 MHz Ti:Sapphire oscillator. The seed beam was delayed with an optical delay line before the 1 kHz amplifier to accommodate for the 100 fs – 14.7 ns time delays between pump and probe. To go beyond 14.7 ns and up to 100 µs, subsequent seed pulses are selected from the 68 MHz seed pulse train accompanied by the appropriate setting of the optical delay line. The polarisation of the excitation beam at sample was set to be at 54.7° with respect to the probe.

For the LIFEtime spectrometer, the pump source was the output of a Yb:KGW amplifier providing 15W, 260 fs pulses at 1030 nm with a 100 kHz repetition rate (Pharos). This was used to drive a BBO-based 515 nm pumped optical parametric amplifier (OPA) to deliver pulses at 355 nm. The pump beam was collimated, travelled over a computer programmable 0 - 16 ns optical delay (1200 mm long, double pass), and focused onto the sample. The pump energy at the sample was attenuated down to 500 nJ and focused down to a 120 × 120 μm^2 spot. The probe source was the output of a Yb:KGW amplifier providing 6 W, 180 fs pulses at 1030 nm with a 100 kHz repetition rate (Pharos). This drove two 3 W BBO/KTA based OPAs. The two Pharos sources share a common 80 MHz oscillator to allow for pump-probe delay steps of 12.5 ns. The probe beam was split to provide probe and reference pulses. To go beyond pump-probe delays of 12.5 ns, subsequent seed pulses were selected from the 80 MHz oscillator. Data were collected using pump-probe delays ranging from 1 ps to 988.5 µs. The probe beams were collimated, synchronised by a fixed optical delay, and focused by a gold parabolic mirror onto the sample. The three beams were overlapped on the sample using a 50 µm pinhole. The probe beams were measured by two separate 128-element detectors. To cover the full spectroscopic window required, data from different detector positions were combined to generate the required spectra.

Solutions of the manganese complexes were prepared at a concentration of *ca*. 2 mmol dm^{-3} in toluene in a thick-walled Duran flask from which light was excluded by covering in aluminium foil. The solution was then flowed (peristaltic pump) through a Harrick cell fitted with a 100 µm Teflon spacer. To ensure that the photoproducts were not themselves irradiated, data were acquired while continuously flowing and rastering the sample. The experiments were performed at ambient temperature which was maintained at 20 ± 1 °C.

Data were initially visualised in the ULTRA View version 2 software,[4] where baseline-correction was undertaken. The resulting spectra were then exported as comma-separated variable files into Origin2022.[5] The spectra were calibrated against samples of polystyrene (200 µm thick) and 1,4-dioxane. Kinetic analysis was performed by fitting to the *expgro*, *expgro2*,*expdec, expdec2* or *expgrodec* functions in Origin2022. All quoted errors are 95 % confidence limits.

2.2 Compound Numbering

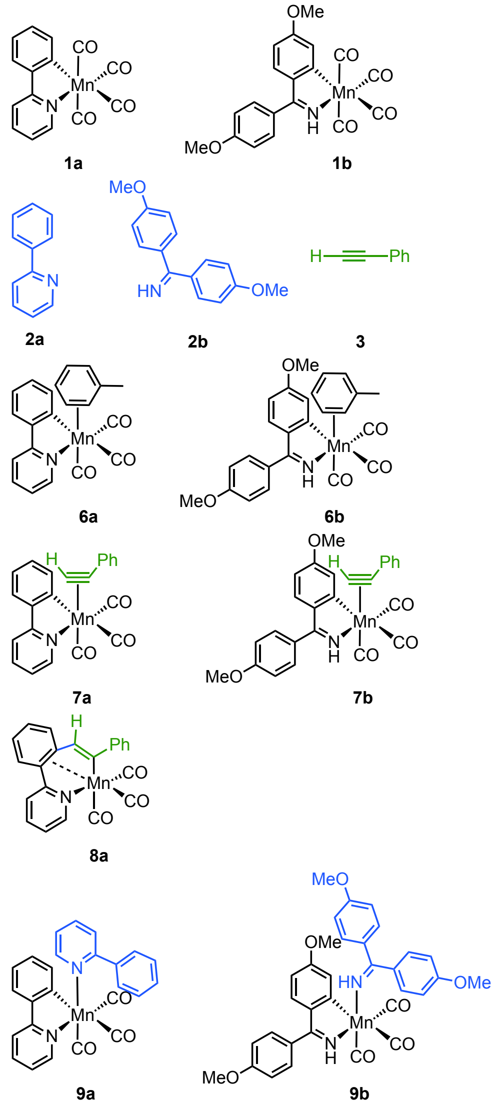

Figure S1 Numbering scheme for compounds reported in the ESI

2.3 Additional TRIR studies on 1a

2.3.1 TRIR study of **1a** in toluene solution with a 2:1 ratio of PhC_2H and 2-ppy.

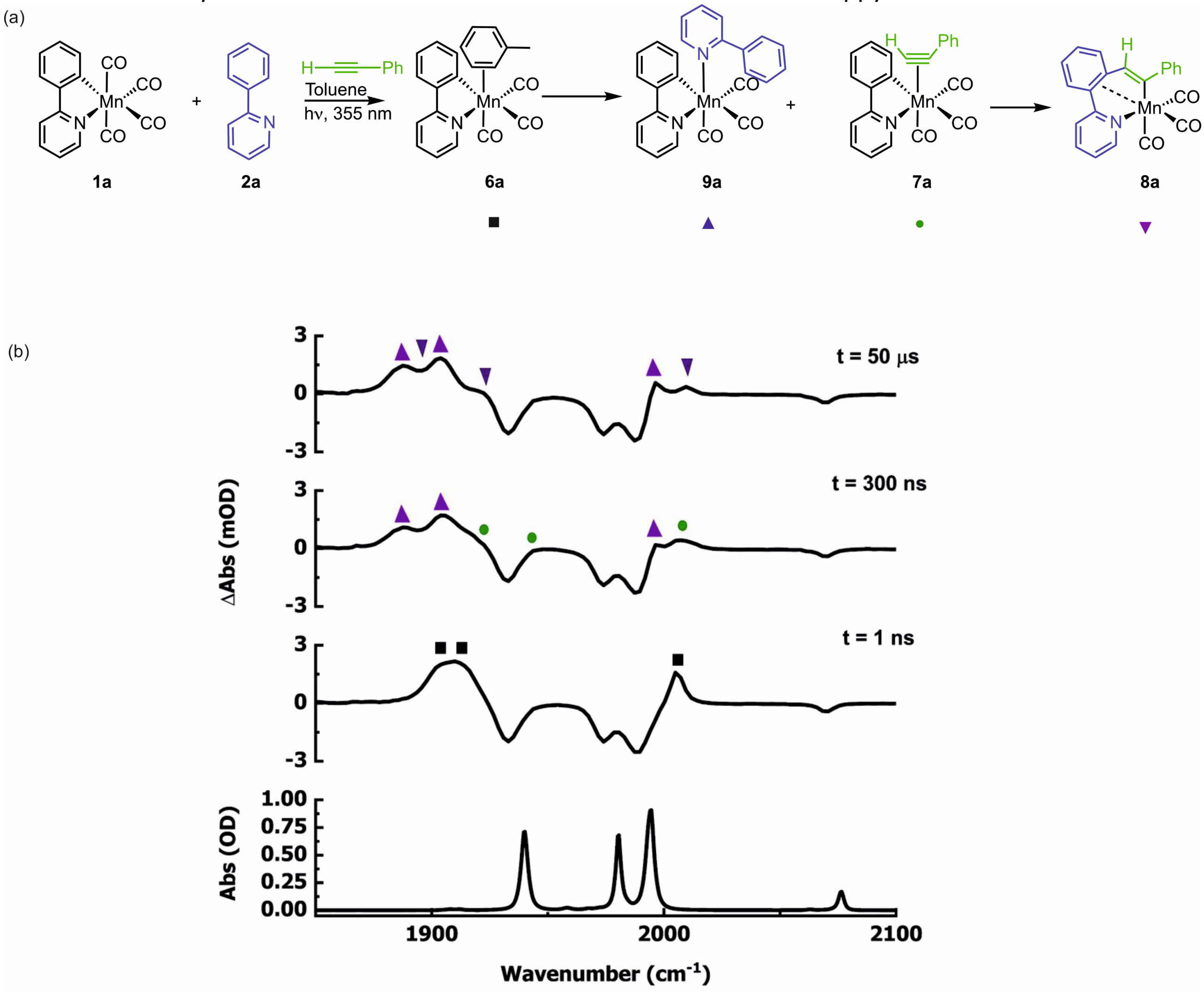

Figure S2 (a) Reaction scheme showing the products formed after photolysis of **1a** in a toluene solution of **2a** and **3**. (b) Bottom ground state IR spectrum of **1a** in toluene solution, above TRIR spectra of **1a** in a toluene solution of **2a** and PhC_2H at pump-probe delays of 1 ns, 300 ns and 50 µs. **6a** (black squares), **7a** (green circles), **8a** (inverted purple triangles) **9a** (blue triangles). The concentrations used in these experiments were [**1a**] 1.98 mmol dm^{-3}, [**2a**] 12.9 mmol dm^{-3} and [**3**] 19.5 mmol dm^{-3}.

2.3.2 TRIR study of **1a** in toluene solution with a 1:1 ratio of PhC_2H and 2-ppy.

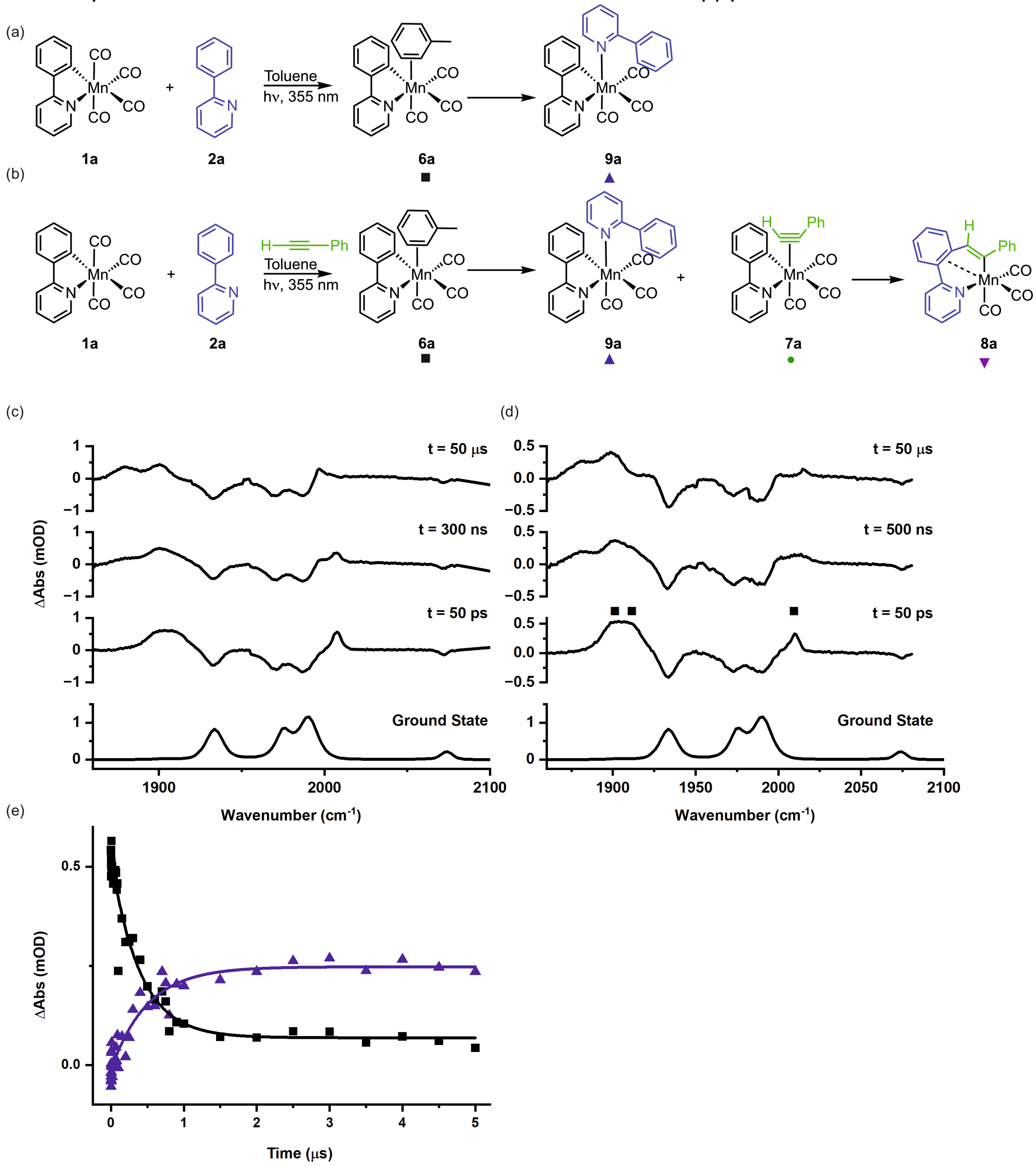

Figure S3 (a) Reaction scheme showing the products formed after photolysis of **1a** in a toluene solution of **2a** (b) Reaction scheme showing the products formed after photolysis of **1a** in a toluene solution of **2a** and **3**. (c) Bottom ground state IR spectrum of **1a** in toluene solution, above TRIR spectra of **1a** in a toluene solution of **2a** at pump-probe delays of 1 ns, 300 ns and 1 μs. (d) Bottom, ground state IR spectrum of **1a** in toluene solution, above TRIR spectra of **1a** in a toluene solution of **2a** and **3** at pump-probe delays of 1 ns, 500 ns and 50 μs. (e) Kinetic plot showing the change in intensity of **6a** (black squares) and **9a** (blue triangles) for a **1a** in a toluene solution of **2a**. The lines show fits to exponential decay (for **6a** $k = (2.52 \pm 0.57) \times 10^6$ s^{-1} $R^2 = 0.95$) and growth functions (for **9a** $k = (2.01 \pm 0.58) \times 10^6$ s^{-1} $R^2 = 0.92$). The concentrations used in these experiments were [**1a**] 2.04 mmol dm^{-3}, [**2a**] 20.5 mmol dm^{-3} and [**3**] 30.9 mmol dm^{-3}.

2.3.3 TRIR study of **1a** in toluene solution with a 1:1 ratio of PhC_2H and 2-ppy.

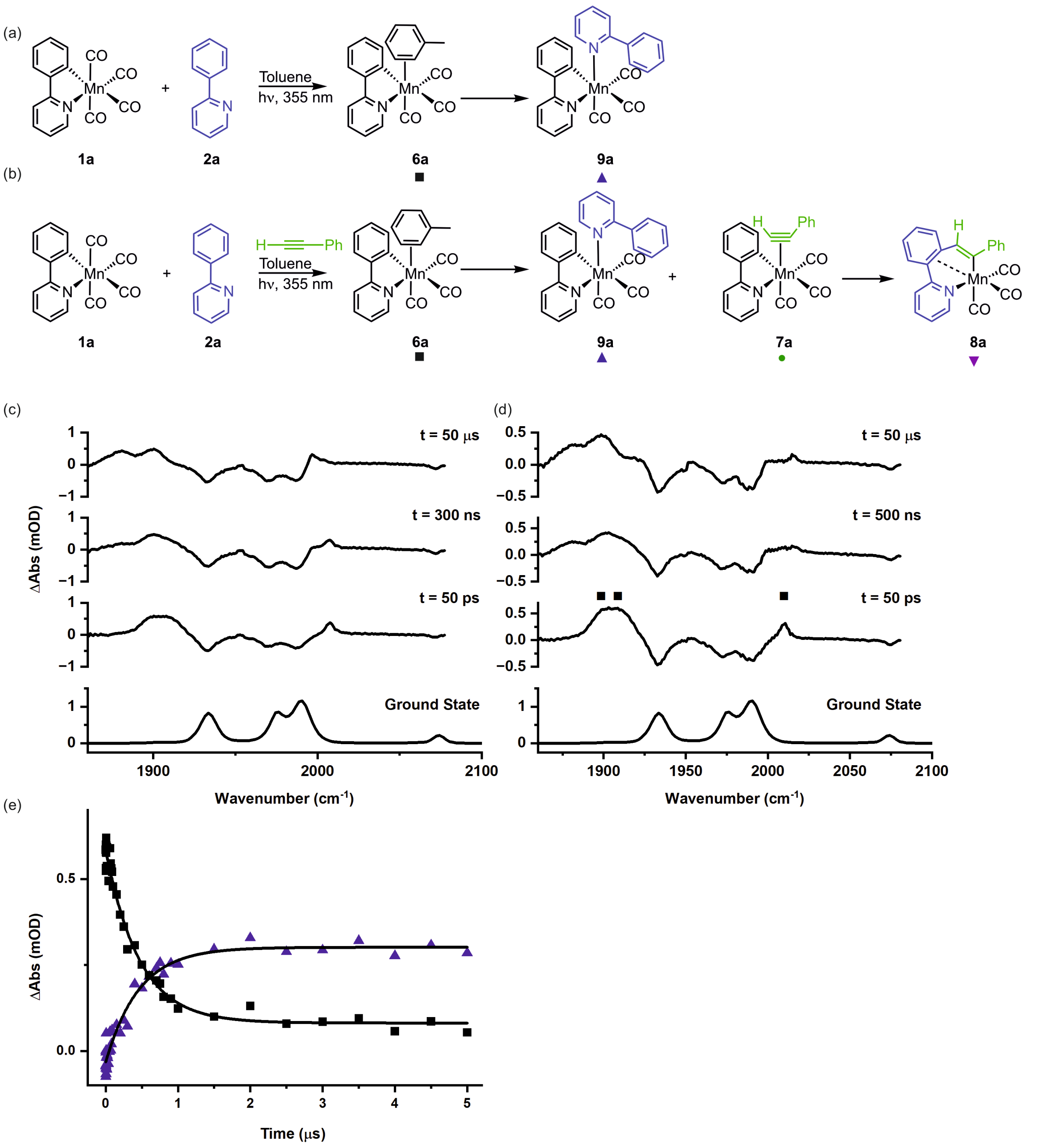

Figure S4 (a) Reaction scheme showing the products formed after photolysis of **1a** in a toluene solution of **2a** (b) Reaction scheme showing the products formed after photolysis of **1a** in a toluene solution of **2a** and **3**. (c) Bottom ground state IR spectrum of **1a** in toluene solution, above TRIR spectra of **1a** in a toluene solution of **2a** at pump-probe delays of 1 ns, 300 ns and 1 μs. (d) Bottom, ground state IR spectrum of **1a** in toluene solution, above TRIR spectra of **1a** in a toluene solution of **2a** and **3** at pump-probe delays of 1 ns, 500 ns and 50 μs. (e) Kinetic plot showing the change in intensity of **6a** (black squares) and **9a** (blue triangles) for a **1a** in a toluene solution of **2a**. The lines show fits to exponential decay (for **6a** k = (2.16 ± 0.29) × 10^6 s^{-1} R^2 = 0.98) and growth functions (for **9a** k = (2.17 ± 0.48) × 10^6 s^{-1} R^2 = 0.96). The concentrations used in these experiments were [**1a**] 2.04 mmol dm^{-3}, [**2a**] 20.5 mmol dm^{-3} and [**3**] 30.9 mmol dm^{-3}.

2.4 Additional TRIR studies on 1b.

2.4.1 TRIR study of **1b** in toluene solution – 1.

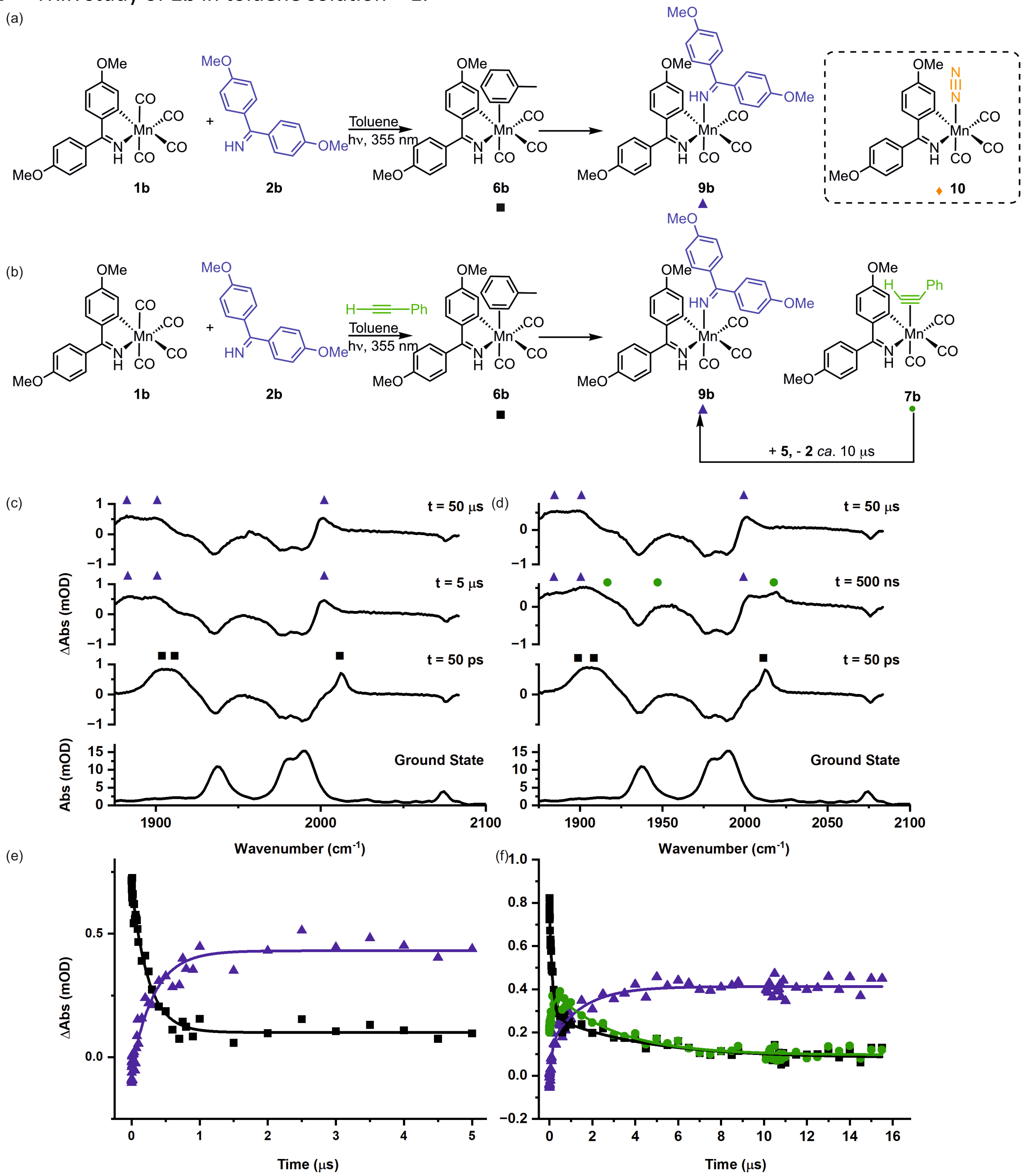

Figure S5 (a) Reaction scheme showing the products formed after photolysis of **1b** in a toluene solution of **2b**. (b) Reaction scheme showing the products formed after photolysis of **1b** in a toluene solution of **2b** and **3**. (c) Bottom, ground state IR spectrum of **1b** in toluene solution, above TRIR spectra of **1b** in a toluene solution of **2b** at pump-probe delays of 50 ps, 5 μs and 50 μs. (d) Bottom, ground state IR spectrum of **1b** in toluene solution, above TRIR spectra of **1b** in a toluene solution of **2b** and **3** at pump-probe delays of 50 ps, 500 ns and 50 μs. (e) Kinetic plot showing the change in intensity of **6b** (black squares) and **9b** (blue triangles) for a **1b** in a toluene solution of **2b**. The lines show fits to exponential decay (for **6b** $k = (4.10 \pm 0.56) \times 10^6$ s^{-1} $R^2 = 0.98$) and growth functions (for **9b** $k = (3.04 \pm 0.73) \times 10^6$ s^{-1} $R^2 = 0.97$). (f) Kinetic plot showing the change in intensity of **6b** (black squares), **7b** (green circles) and **9b** (blue triangles) for **1b** in a toluene solution of **2b** and **3**. In this data set, the data with pump-probe delays between 9999.85 μs and 10100 μs were removed

due artefacts arising from the pump pulse. The lines show fits to biexponential decay (for **6b** k_1 = (7.46 ± 1.21) × 10^6 s^{-1}, k_2 = (2.34 ± 1.15) × 10^5 s^{-1}, R^2 = 0.99), biexponential growth (for **9b** k_1 = (8.69 ± 6.25) × 10^6 s^{-1}, k_2 = (6.83 ± 3.-4) × 10^5 s^{-1}, R^2 = 0.97) and exponential growth and decay (for **7b** k_1 = (6.62 ± 4.60) × 10^6 s^{-1}, k_2 = (3.50 ± 0.77) × 10^5 s^{-1}, R^2 = 0.95). The concentrations used in these experiments were [**1b**] 2.03 mmol dm^{-3}, [**2b**] 20.4 mmol dm^{-3} and [**3**] 30.9 mmol dm^{-3}.

2.4.2 TRIR study of **1b** in toluene solution – 2.

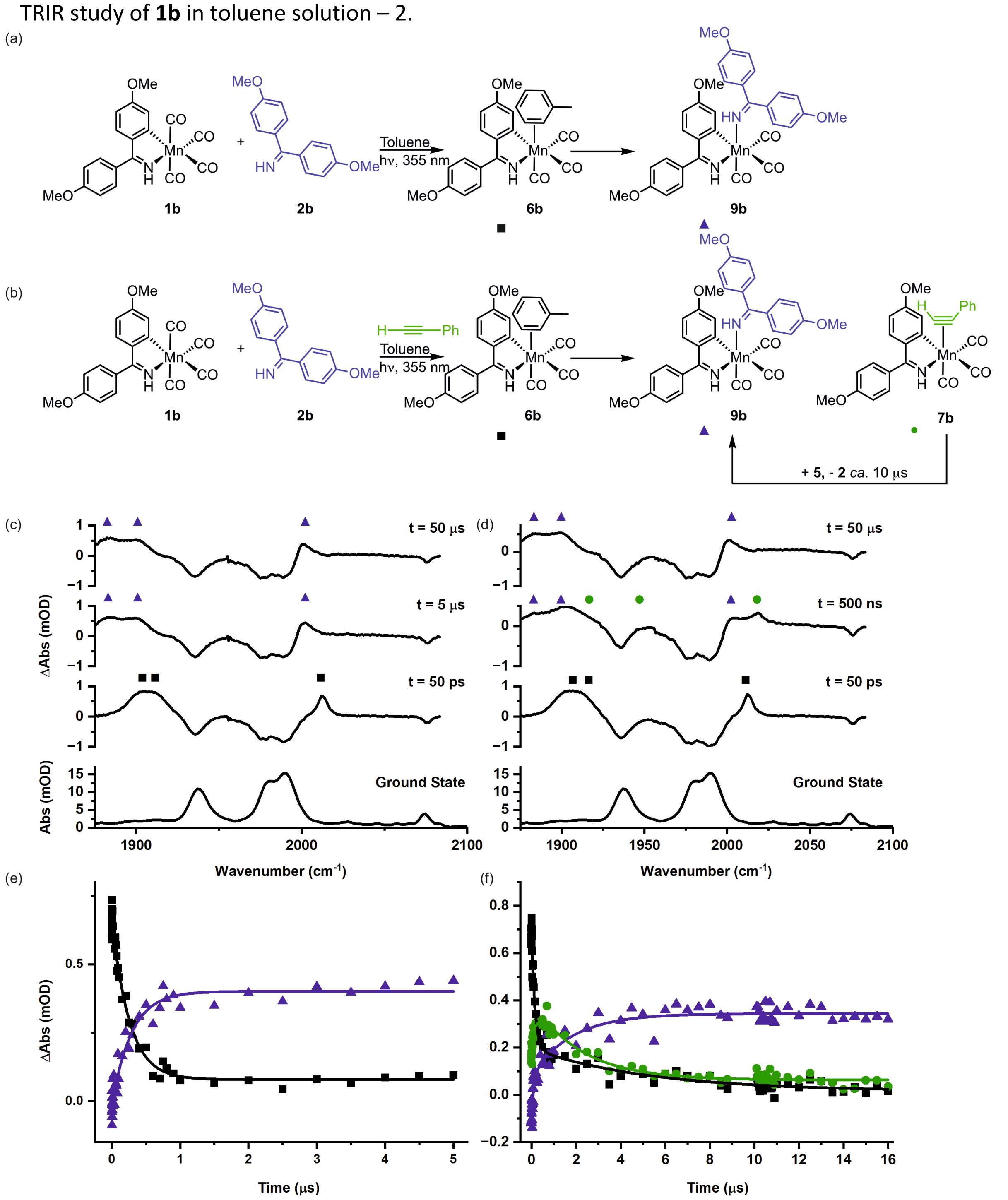

Figure S6 (a) Reaction scheme showing the products formed after photolysis of **1b** in a toluene solution of **2b**. (b) Reaction scheme showing the products formed after photolysis of **1b** in a toluene solution of **2b** and **3**. (c) Bottom, ground state IR spectrum of **1b** in toluene solution, above TRIR spectra of **1b** in a toluene solution of **2b** at pump-probe delays of 50 ps, 5 ms and 50 μs. (d) Bottom, ground state IR spectrum of **1b** in toluene solution, above TRIR spectra of **1b** in a toluene solution of **2b** and **3** at pump-probe delays of 50 ps, 500 ns and 50 μs. (e) Kinetic plot showing the change in intensity of **6b** (black squares) and **9b** (blue triangles) for a **1b** in a toluene solution of **2b**. The lines show fits to exponential decay (for **6b** $k = (3.93 \pm 0.54) \times 10^6\ s^{-1}$ $R^2 = 0.98$) and growth functions (for **9b** $k = (3.48 \pm 0.90) \times 10^6\ s^{-1}$ $R^2 = 0.95$). (f) Kinetic plot showing the change in intensity of **6b** (black squares), **7b** (green circles) and **9b** (blue triangles) for **1b** in a toluene solution of **2b** and **3**. In this data set, the data with pump-probe delays between 9999.85 μs and 10100 μs were removed due artefacts arising from the pump pulse. The lines show fits to biexponential decay (for **6b** $k_1 = (6.79 \pm 1.26)$

× 10^6 s^{-1}, k_2 = (1.88 ± 1.25) × 10^5 s^{-1}, R^2 = 0.99), biexponential growth (for **9b** k_1 = (9.95 ± 7.68) × 10^6 s^{-1}, k_2 = (5.58 ± 2.89) × 10^5 s^{-1}, R^2 = 0.95) and exponential growth and decay (for **7b** k_1 = (1.07 ± 0.53) × 10^7 s^{-1}, k_2 = (4.52 ± 1.17) × 10^5 s^{-1}, R^2 = 0.93). The concentrations used in these experiments were [**1b**] 2.03 mmol dm^{-3}, [**2b**] 20.4 mmol dm^{-3} and [**3**] 30.9 mmol dm^{-3}.

2.4.3 Collated Rate Constants

A series of technical repeats were performed to ensure reproducibility in the difference in behaviour between the 2-phenylpyridine and 1,1-bis(4-methoxyphenyl)methanimine-based systems (Figure S3-S6). In each case, regardless of the concentrations used, the qualitative observation that the competitive formation of the respective complexes **7** and **9** from **6** occurred in all cases. In the case of the 2-phenylpyridine system, then **7a** proceeded to undergo migratory insertion to give 8a, while 9a remained unchanged. In the 1,1-bis(4-methoxyphenyl)methanimine case, then **7b** was transformed to **9b** with no evidence of the migratory insertion taking place. Furthermore, the difference between the pseudo first order rate constants obtained under identical conditions were generally within 95% confidence limits between experiments. (Tables S1-3).

The kinetics for the experiments involving **1b**, **2b** and PhC_2H were modelled as follows. The loss of the band due to the initially formed toluene complex **6b** was fitted to biexponential kinetics as there was a small overlap between this band and that for **7b** which was subsequently also lost. The band for **9b** was modelled with a biexponential growth function as it was formed in a fast step (substitution of the toluene in **6b** by **2b**) then a slow step(displacement of the coordinated alkyne in **7b** also by **2b**). The formation of **7b** from **6b** and the subsequent substitution of the alkyne by **2b** was modelled with an exponential growth and decay function.

Table S1 . Observed first order rate constants for the substitution of toluene by **2a** for **6a** in toluene solution

Figure	Loss of **6a** / s^{-1}	Formation of **9a** / s^{-1}
S3	$(2.52 \pm 0.57) \times 10^6$	$(2.01 \pm 0.58) \times 10^6$
S4	$(2.16 \pm 0.29) \times 10^6$	$(2.17 \pm 0.48) \times 10^6$

Table S2 . Observed first order rate constants for the substitution of toluene by **2b** for **6b** in toluene solution.

Figure	Loss of **6b** / s^{-1}	Formation of **9b** / s^{-1}
S5	$(3.04 \pm 0.73) \times 10^6$	$(4.10 \pm 0.56) \times 10^6$
S6	$(3.48 \pm 0.90) \times 10^6$	$(3.93 \pm 0.54) \times 10^6$

Table S3 . Observed first order rate constants for the conversion between **6b**, **7b**, and **9b**.

Figure	Fast loss of **6b** / 10^6 s^{-1}	Slow loss of **6b** / 10^5 s^{-1}	Fast growth of **9b** / 10^6 s^{-1}	Slow growth of **9b** / 10^5 s^{-1}	Growth of **7b** / 10^6 s^{-1}	Loss of **7b** / 10^5 s^{-1}
3	6.98 ± 0.27	3.77 ± 0.74	6.11 ± 0.46	3.11 ± 0.21	9.69 ± 2.87	3.47 ± 0.19
S5	7.46 ± 1.21	2.34 ± 1.15	8.96 ± 6.25	6.65 ± 3.13	6.62 ± 4.60	3.50 ± 0.77
S6	6.79 ± 1.26	1.88 ± 1.25	9.95 ± 7.68	5.58 ± 2.89	10.7 ± 5.3	4.52 ± 1.17

3 DFT Calculations

3.1 Methodology

Calculations to determine the relative energies of intermediates and transition states were performed using the TURBOMOLE V6.4 package using the resolution of identity (RI) approximation.[6] Initial optimisations were performed at the (RI-)BP86/SV(P) level, followed by frequency calculations at the same level. All minima were confirmed as such by the absence of imaginary frequencies. Single-point energies were then performed on the (RI-)BP86/SV(P) optimised geometries using the hybrid PBE0 functional and the flexible def2-TZVPP basis set. Solvation effects were modelled using COSMO[7] using the dielectric constant of 2.38 for toluene and energies were corrected for dispersion using Grimme's D3-method with Becke-Johnson dampening. Energies are reported in Table S4 and xyz coordinates collated in the attached file.

In the case of the complexes containing *N*-bound ligands, **9a** and **9b**, three different arrangements of the κ^1-bound ligand were investigated (Figure S7). In both cases, the lowest energy conformation is the one with the κ^1-ligand orientated to position a phenyl substituent over the tricarbonyl group as this, presumably, has less of a steric clash than the alternative geometries with the ligand over the cyclomanganated group. In the manuscript, only the lowest energy form is reported.

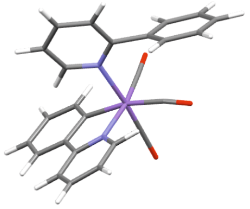

9a
-35

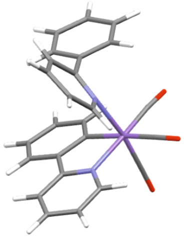

9a-1
-31

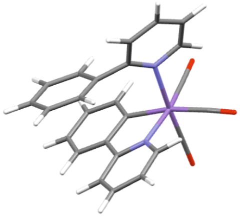

9a-2
-25

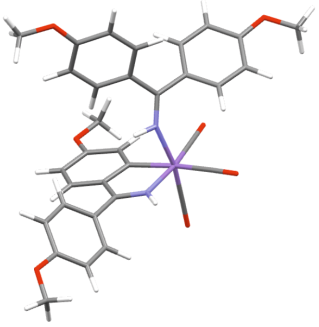

9b
-80

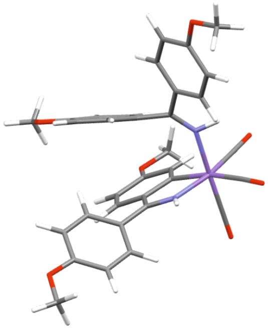

9b-1
-68

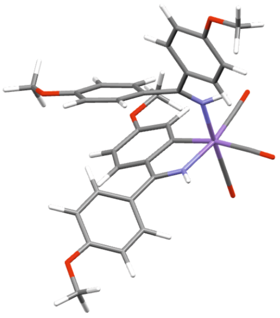

9b-2
-68

Figure S7 Calculated conformations of the κ^1-bound ligands in complexes **9a** and **9b**. Energies are Gibbs energies at 298 K in kJ mol^{-1} at the D3-pbe0/def2-TZVPP//bp86/SV(P) level of theory with COSMO solvent correction in toluene for the corresponding isodesmic reactions shown in Figure 4 of the manuscript.

Table S4. Calculated energies for the states reported

Level of theory	(RI)-bp86/SV(P)			(RI)-pbe0/def2-TZVPP		
	SCF Energy<br>Hartrees	ZPE Energy<br>Hartrees	Chemical Potential<br>kJ mol^{-1}	SCF Energy<br>Hartrees	SCF Energy COSMO Toluene<br>Hartrees	D3 Correction<br>Hartrees
**2a**	-479.004597	0.165117	340.21	-478.9366917	-478.9411698	-0.021666
**2b**	-785.237646	0.261246	568.38	-785.167605	-785.1755729	-0.034736
**3**	-308.169892	0.106496	199.26	-308.123224	-308.127012	-0.012246
**6a**	-2240.684022	0.306464	652.08	-2240.255273	-2240.262014	-0.062953
**6b**	-2546.926341	0.402966	884.33	-2546.492974	-2546.502421	-0.075306
**7a**	-2277.516190	0.289777	615.66	-2277.070641	-2277.077865	-0.061107
**7b**	-2583.758694	0.385838	840.51	-2583.308834	-2583.318622	-0.072291
**8a**	-2277.545461	0.291608	622.44	-2277.104429	-2277.112473	-0.064859
**8b**	-2583.785980	0.387530	845.24	-2583.339567	-2583.350311	-0.074683
**9a**	-2448.344807	0.348335	760.86	-2447.882431	-2447.891502	-0.074128
**9a-1**	-2448.340800	0.348426	759.69	-2447.878379	-2447.887392	-0.076362
**9a-2**	-2448.336935	0.348329	760.29	-2447.875086	-2447.883547	-0.078335
**9b**	-3060.842415	0.541076	1213.49	-3060.842415	-3060.383175	-0.096943
**9b-1**	-3060.833535	0.541193	1215.08	-3060.361381	-3060.374543	-0.101558
**9b-2**	-3060.833535	0.541191	1215.15	-3060.361378	-3060.374542	-0.101573
Toluene	-271.360952	0.124206	245.59	-271.326186	-271.328614	-0.012093

4 References

(1) Bruce, M. I.; Goodall, B. L.; Matsuda, I. Cyclometallation reactions .13. Reactions of phenyl-substituted heterocyclic nitrogen-donor ligands. *Aust. J. Chem.* **1975**, *28*, 1259-1264.

(2) He, R.; Huang, Z.-T.; Zheng, Q.-Y.; Wang, C. Manganese-catalyzed dehydrogenative [4+2] annulation of N–H imines and alkynes by C–H/N–H activation. *Angew. Chem. Int. Ed.* **2014**, *53*, 4950-4953.

(3) Greetham, G. M.; Donaldson, P. M.; Nation, C.; Sazanovich, I. V.; Clark, I. P.; Shaw, D. J.; Parker, A. W.; Towrie, M. A 100 kHz time-resolved multiple-probe femtosecond to second infrared absorption spectrometer. *Appl. Spectrosc.* **2016**, *70*, 645-653.

(4) *Ultra view data analysis*; STFC: 2012.

(5) *Originpro*; OriginLab Corporation: Northampton, MA, USA.

(6) (a) Császár, P.; Pulay, P. Geometry optimization by direct inversion in the iterative subspace. *J. Mol. Struct.* **1984**, *114*, 31-34. (b) Ahlrichs, R.; Bär, M.; Häser, M.; Horn, H.; Kölmel, C. Electronic structure calculations on workstation computers: The program system turbomole. *Chem. Phys. Lett.* **1989**, *162*, 165-169. (c) Deglmann, P.; Furche, F.; Ahlrichs, R. An efficient implementation of second analytical derivatives for density functional methods. *Chem. Phys. Lett.* **2002**, *362*, 511-518. (d) Deglmann, P.; May, K.; Furche, F.; Ahlrichs, R. Nuclear second analytical derivative calculations using auxiliary basis set expansions. *Chem. Phys. Lett.* **2004**, *384*, 103-107. (e) Eichkorn, K.; Treutler, O.; Öhm, H.; Häser, M.; Ahlrichs, R. Auxiliary basis sets to approximate coulomb potentials. *Chem. Phys. Lett.* **1995**, *242*, 652-660. (f) Eichkorn, K.; Weigend, F.; Treutler, O.; Ahlrichs, R. Auxiliary basis sets for main row atoms and transition metals and their use to approximate coulomb potentials. *Theor. Chem. Acc.* **1997**, *97*, 119-124. (g) Treutler, O.; Ahlrichs, R. Efficient molecular numerical integration schemes. *J. Chem. Phys.* **1995**, *102*, 346-354. (h) von Arnim, M.; Ahlrichs, R. Geometry optimization in generalized natural internal coordinates. *J. Chem. Phys.* **1999**, *111*, 9183-9190.

(7) Klamt, A.; Schuurmann, G. Cosmo: A new approach to dielectric screening in solvents with explicit expressions for the screening energy and its gradient. *J. Chem. Soc., Perkin Trans. 2* **1993**, 799-805.
